# Tanshinone IIA suppresses the progression of lung adenocarcinoma through regulating CCNA2-CDK2 complex and AURKA/PLK1 pathway

**DOI:** 10.1038/s41598-021-03166-2

**Published:** 2021-12-08

**Authors:** Ziheng Li, Ying Zhang, Yuan Zhou, Fuqian Wang, Chao Yin, Li Ding, Shunbo Zhang

**Affiliations:** Faculty of Pharmacy, Hubei University of Chinese Medicine, Wuhan, 430065 China

**Keywords:** Cancer, Computational biology and bioinformatics

## Abstract

Lung adenocarcinoma (LUAD) belongs to a subgroup of non-small cell lung cancer (NSCLC) with an increasing incidence all over the world. Tanshinone IIA (TSA), an active compound of Salvia miltiorrhiza Bunge., has been found to have anti-tumor effects on many tumors, but its anti-LUAD effect and its mechanism have not been reported yet. In this study, bio-information analysis was applied to characterize the potential mechanism of TSA on LUA, biological experiments were used to verify the mechanisms involved. TCGA, Pubchem, SwissTargetPrediction, Venny2.1.0, STRING, DAVID, Cytoscape 3.7.2, Omicshare, GEPIA, RSCBPDB, Chem Draw, AutoDockTools, and PyMOL were utilized for analysis in the bio-information analysis and network pharmacology. Our experiments in vitro focused on the anti-LUAD effects and mechanisms of TSA on LUAD cells (A549 and NCI-H1975 cells) via MTT, plate cloning, Annexin V-FITC and PI dual staining, flow cytometry, and western blot assays. A total of 64 differentially expressed genes (DEGs) of TSA for treatment of LUAD were screened out. Gene ontology and pathway analysis revealed characteristic of the DEGs network. After GEPIA-based DEGs confirmation, 46 genes were considered having significant differences. Further, 10 key DEGs (BTK, HSD11B1, ADAM33, TNNC1, THRA, CCNA2, AURKA, MIF, PLK1, and SORD) were identified as the most likely relevant genes from overall survival analysis. Molecular Docking results showed that CCNA2, CDK2 and PLK1 had the lowest docking energy. MTT and plate cloning assays results showed that TSA inhibited the proliferation of LUAD cells in a concentration-dependent manner. Annexin V-FITC and PI dual staining and flow cytometry assays results told that TSA promoted the apoptosis of the two LUAD cells in different degrees, and induced cycle arrest in the G1/S phase. Western blot results showed that TSA significantly down-regulated the expression of CCNA2, CDK2, AURKA, PLK1, and p-ERK. In summary, TSA could suppress the progression of LUAD by inducing cell apoptosis and arresting cell cycle, and these were done by regulating CCNA2-CDK2 complex and AURKA/PLK1 pathway. These findings are the first to demonstrate the molecular mechanism of TSA in treatment of LUAD combination of network bio-information analysis and biological experiments in vitro.

## Introduction

Lung cancer is a malignant tumor in which cancer cells originate from bronchial mucosa epithelium^1^. At present, lung cancer has become the world's highest incidence rate and most death cancer^2^. Lung cancer is generally divided into two types, namely small cell lung cancer and NSCLC, the latter of which accounts for 85%^3^. NSCLC is subdivided into squamous cell carcinoma, adenocarcinoma, and large cell carcinoma. LUAD is the most a common type of lung cancer, accounting for about 35% of all lung cancers^4^. Patients with early LUAD can have their tumors removed surgically, but most patients are diagnosed with advanced lung cancer, so they had to be treated conservatively with anti-cancer drugs^5^. Several useful anti-cancer drugs have been found for patients with LUAD, such as Gemcitabine^6^. Although these drugs are effective in treating lung cancer, they are also associated with significant side effects in humans, for example, has an inhibitory effect on human bone marrow while treating lung cancer, some patients have anemia, leukopenia and thrombocytopenia after taking the drug^7^. Therefore, new treatments for LUAD with less side effects and screening new effective targets for LUAD are needed.

Cyclins are important regulators of cell division. During the cell cycle, Cyclins bind to Cyclin-dependent kinases (CDKs) to form an active complex that catalyzes substrate phosphorylation, thus promoting the orderly cell cycle^8^. Among them, CDK2 binds to Cyclin A2 (CCNA2) to form a proteasome complex that regulates the transition from G1 to S phase of the cell cycle and the progression of S phase^9^. Studies have shown that overexpression of CCNA2-CDK2 complex is closely associated with the occurrence of lung cancer, stomach cancer, leukemia, breast cancer and other tumors^10^. Therefore, it is of great significance to find new CCNA2-CDK2 complex inhibitors for the treatment of LUAD.

TSA is a lipophilic component isolated from traditional Chinese medicine Salvia miltiorrhiza Bunge. TSA has been proved to have a variety of pharmacological activities, such as anti-inflammation, anti-oxidant and anti-tumor activities^11^. In recent years, research has shown that TSA has a cytotoxic effect on a series of tumor cells, Liu et al. found that TSA alleviated colorectal tumorigenesis through inhibition of intestinal inflammation^12^. Wang et al. found that TSA inhibited cell viability of nasopharyngeal carcinoma through regulating microRNA‑125b/foxp3/caspase‑1 signaling^13^. However, the role of TSA against LUAD remains unknown. In this study, we explored the mechanism of TSA in LUAD by using the biological information analysis approach and biological experimental in vitro and in vivo, to provide a theoretical basis for further development of TSA.

## Results

### Collection of TSA-related DEGs

**Figure 1:**
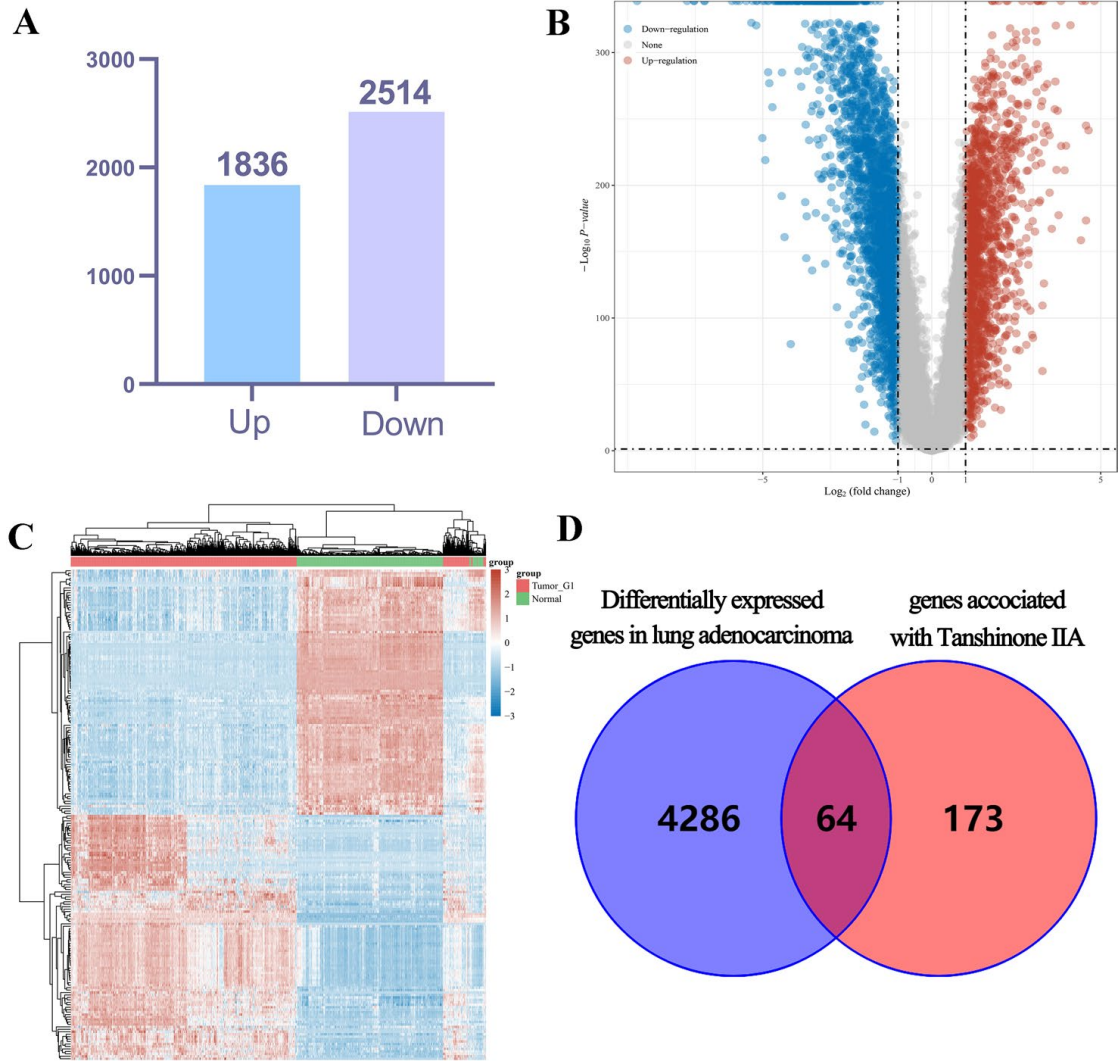
Identification of the differentially expressed genes (DEGs) of LUAD in the TCGA cohort. (**A**) The DEGs between tumors and normal tissues. (**B**) Volcano plots, to Visualize the DEGs. (**C**) The expression trend of 4350 DEGs shown as heatmap. (**D**) Venn diagram to identify TSA-related DEGs between tumor and adjacent normal tissue.

A total of 4350 DEGs were identified and shown in volcano plots, including 1836 up-regulated and 2514 down-regulated (Fig. 1A,B). The expression of these 4350 genes in the TCGA data set (including 1014 cancerous tissues and 686 normal tissues) is shown in the heatmap (Fig. 1C). Among the 4350 DEGs, there were 64 intersections with TSA-related targets (Fig. 1D), these 64 DEGs were looked as the target genes of TSA for treatment of LUAD, and were shown in supplementary Table S1.

### PPI Network Construction and Functional Enrichment Analysis of TSA-related DEGs

**Figure 2:**
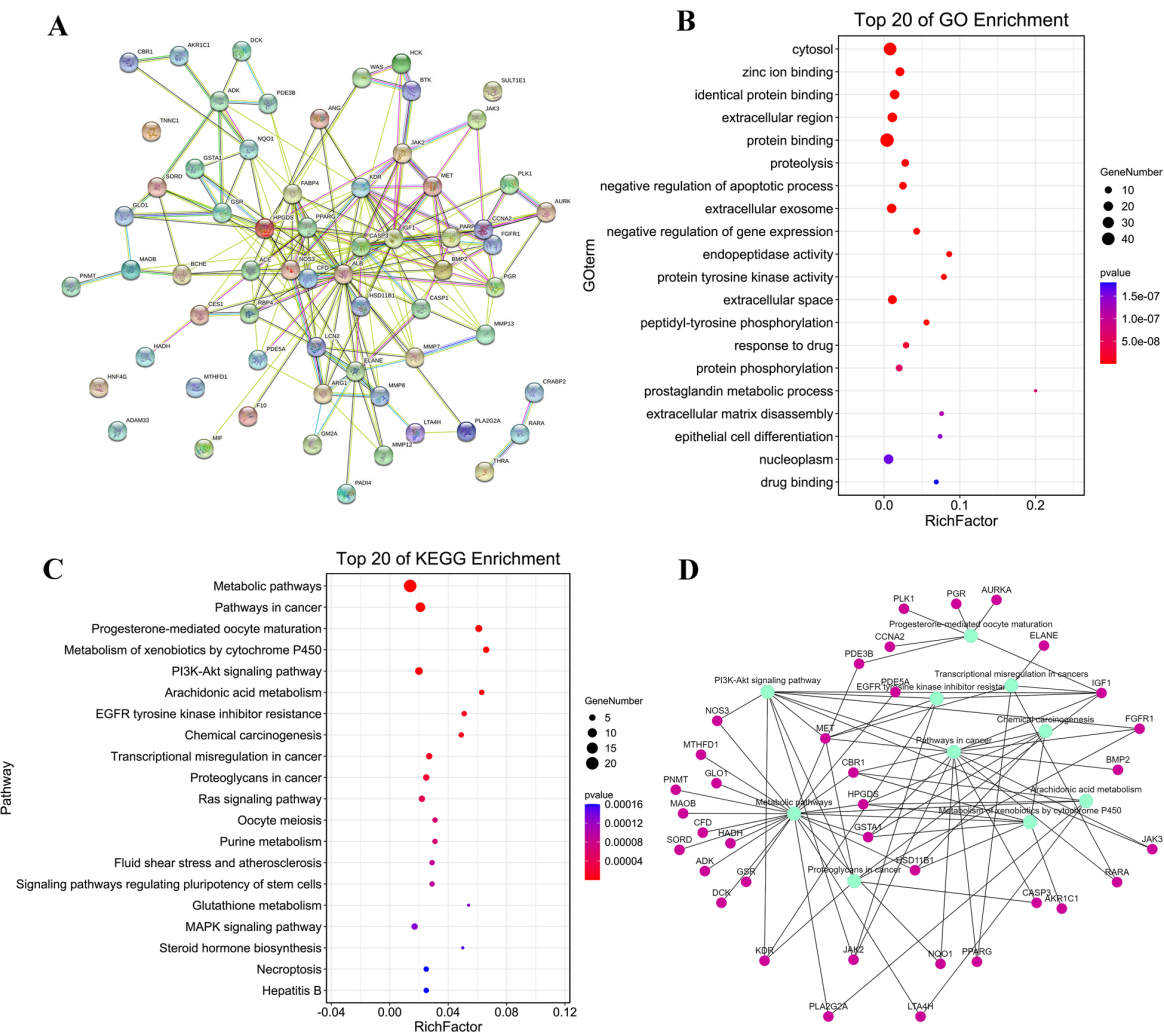
PPI network, GO and KEGG analyses of all DEGs related TSA. (**A**) PPI network constructed by STRING. (**B**) Go analysis items. (**C**) KEGG pathways. (**D**) Network of interactions between the top 10 pathways and 64 target genes, the green indicates the pathways, the pink indicates the genes.

64 target genes were putted into STRING for PPI network. The network has shown that CASP3, PPARG, ALB, JAK2, BTK, and CCNA2 might be the hub genes. THAR, ADAM33, HNF4G, TNNC1, RARA, and SULT1E1were less likely to be hub genes (Fig. 2A). Then, the 64 genes were imported to DAVID for GO and KEGG enrichment analysis. As shown in Fig. 2B, among the top 20 enrichment results, 5 were CC (25%), 6 were MF (30%), and 9 were BP (45%). CCs included cytosol, extracellular region, extracellular exosome, extracellular space, nucleoplasm. MFs included zinc ion binding, identical protein binding, endopeptidase activity, protein tyrosine kinase activity, drug binding. BPs included proteolysis, negative regulation of apoptotic process, peptidyl-tyrosine phosphorylation, response to drug, protein phosphorylation, prostaglandin metabolic process, extracellular matrix disassembly, epithelial cell differentiation, negative regulation of gene expression. For KEGG pathway analysis, 64 target genes were mainly enriched in metabolic pathways, pathways in cancer, Progesterone-mediated oocyte maturation, arachidonic acid metabolism, PI3K-Akt signaling pathway (Fig. 2C). We then constructed a network of interactions between the top 10 pathways and 64 target genes (Fig. 2D).

### Identification and Overall Survival Analysis of TSA-related DEGs

**Figure 3:**
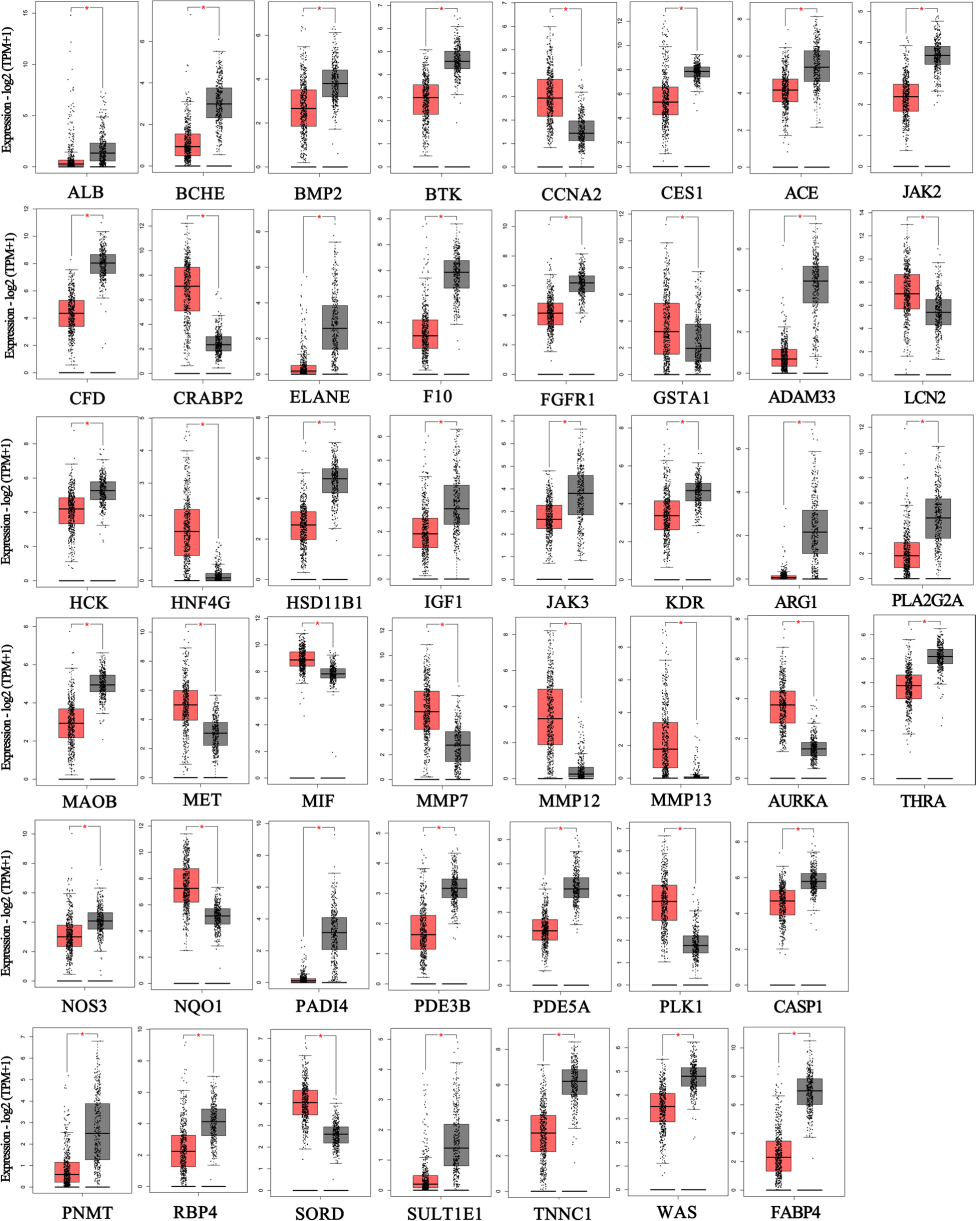
GEPIA analyzed genes that are significantly differentially expressed in LUAD. Red color represents tumor tissue, and black color represents normal tissue.

**Figure 4:**
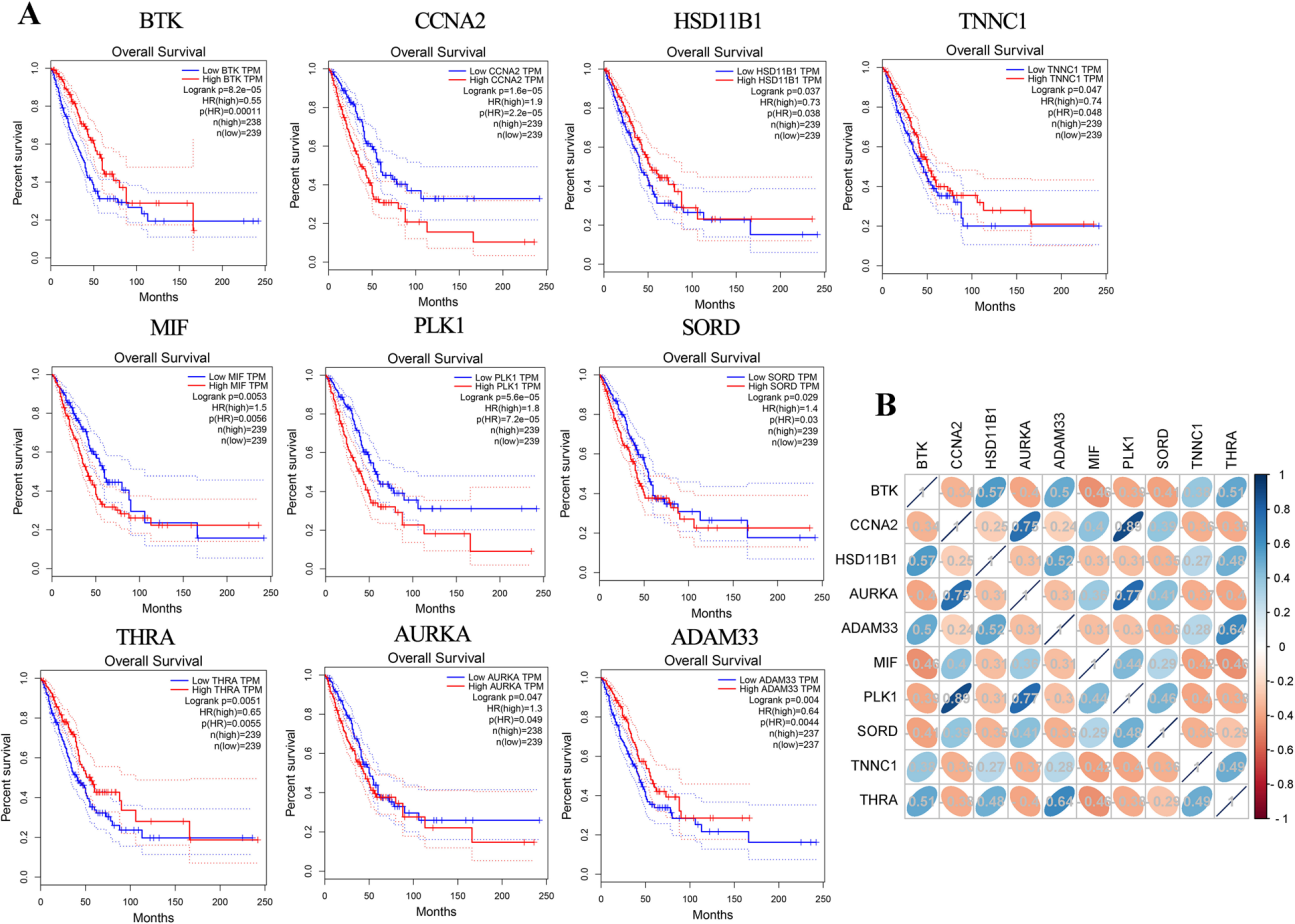
(**A**) Genes analyzed by GEPIA that affect the overall survival of LUAD patients. (**B**) Correlation analysis of 10 genes.

We imported the 64 genes into GEPIA for identification and overall survival analysis for further confirming the accuracy of the analysis of TSA-related DEG. As shown in Fig. 3, there were 46 gens had significant differential expression in LUAD among those 64 gens. We further performed the overall survival analysis of these 46 genes, it was found that low expression of BTK, HSD11B1, ADAM33, TNNC1, and THRA and the high expression of CCNA2, AURKA, MIF, PLK1, and SORD were more likely to encounter LUAD patient death earlier and shorten survival time (Fig. 4A, *P* < 0.05). Further, these 10 genes were performed correlation analysis. From Fig. 4B, CCNA2 and AURKA (correlation = 0.75), CCNA2 and PLK1 (correlation = 0.89), AURKA and PLK1 (correlation = 0.77) were the three pairs of genes with the most positive correlation. BTK and MIF (correlation = -0.46), BTK and SORD (correlation = -0.41), AURKA and THRA (correlation   = − 0.40 were the three pairs of most negatively correlated genes.

### Molecular Docking and Analysis

**Figure 5:**
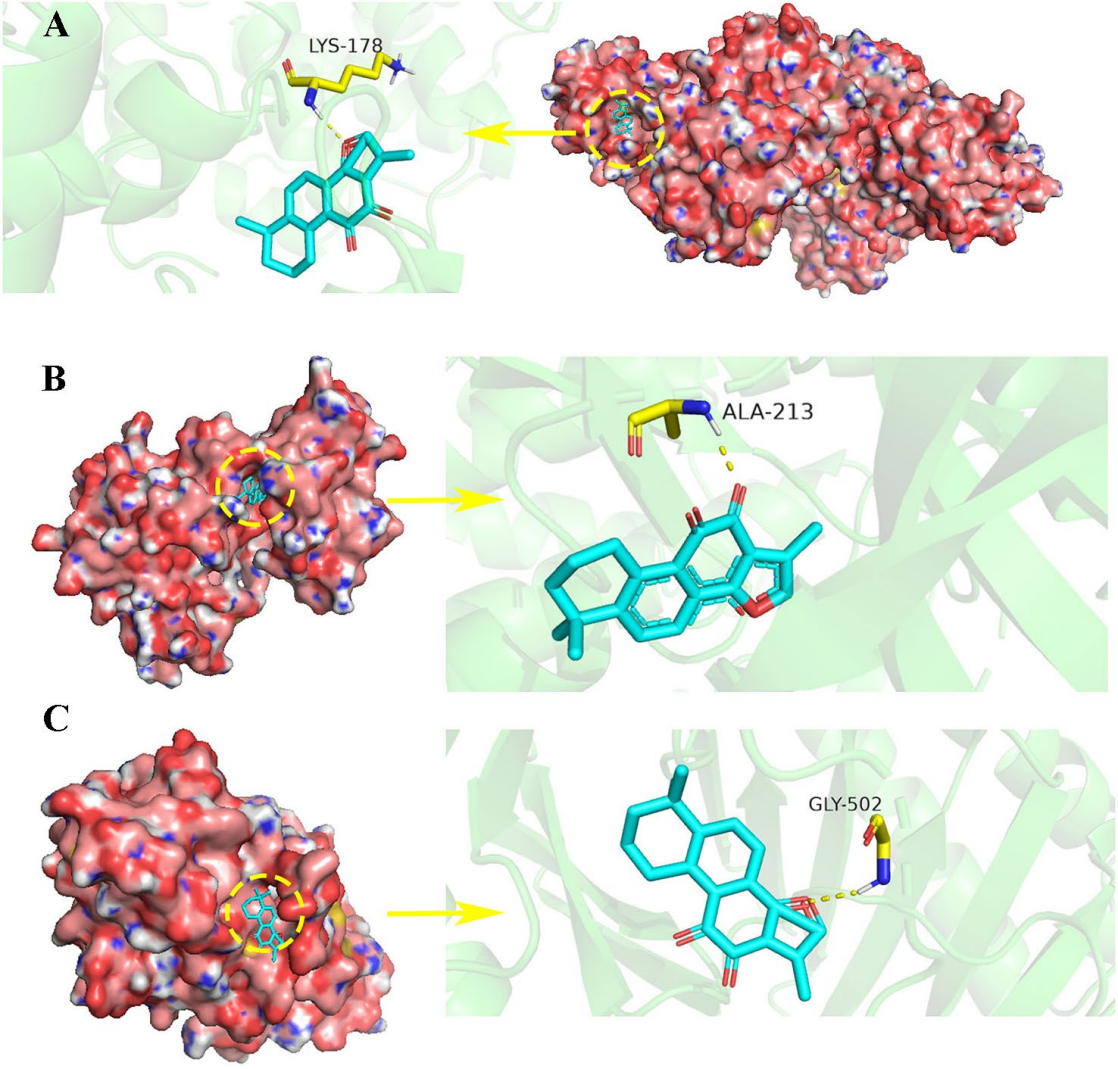
The molecular docking between TSA and corresponding target. (**A**) CCNA2-CDK2 complex-TSA. (**B**) AURKA-TSA. (C) PLK1-TSA.

In order to further verify the possibility of these 10 targets as important targets of TSA against LUAD, we conducted virtual molecular docking between TSA and these 10 genes using AutoDockTools-1.5.6 software. The docking energy and bonds were shown in Table 1, the 3D images and binding sites of top 3 targets were shown in Fig. 5. From results, the 10 targets had strong binding ability with TSA. And it was found that AURKA, PLK1, and CCNA2 had the lower docking energy than the others. This provided a certain basis for our biological experiments.

**Table 1:** Dock binding free energies(^△^Gb) and bonds of the proteins.

Compound	Proteins	^△^Gb(kcal/mol)	Bonds formed between functional groups of components and proteins’ residues	
			Proteins’ residues	bond
TSA	CCNA2-CDK2 complex	− 7.71	A:LYS141	H-bond
	AURKA	− 6.45	A:ALA213	H-bond
	PLK1	− 6.53	A:GLY502	H-bond
	SORD	− 6.4	B:LYS141	H-bond
	HSD11B1	− 5.19	A:GLY470	H-bond
	BTK	− 4.42	A:TRP588	H-bond
	ADAM33	− 5.15	A:HIS379	H-bond
	TNNC1	–	–	–
	THRA	–	–	–

### TSA inhibits the proliferation of LUAD cells

**Figure 6:**
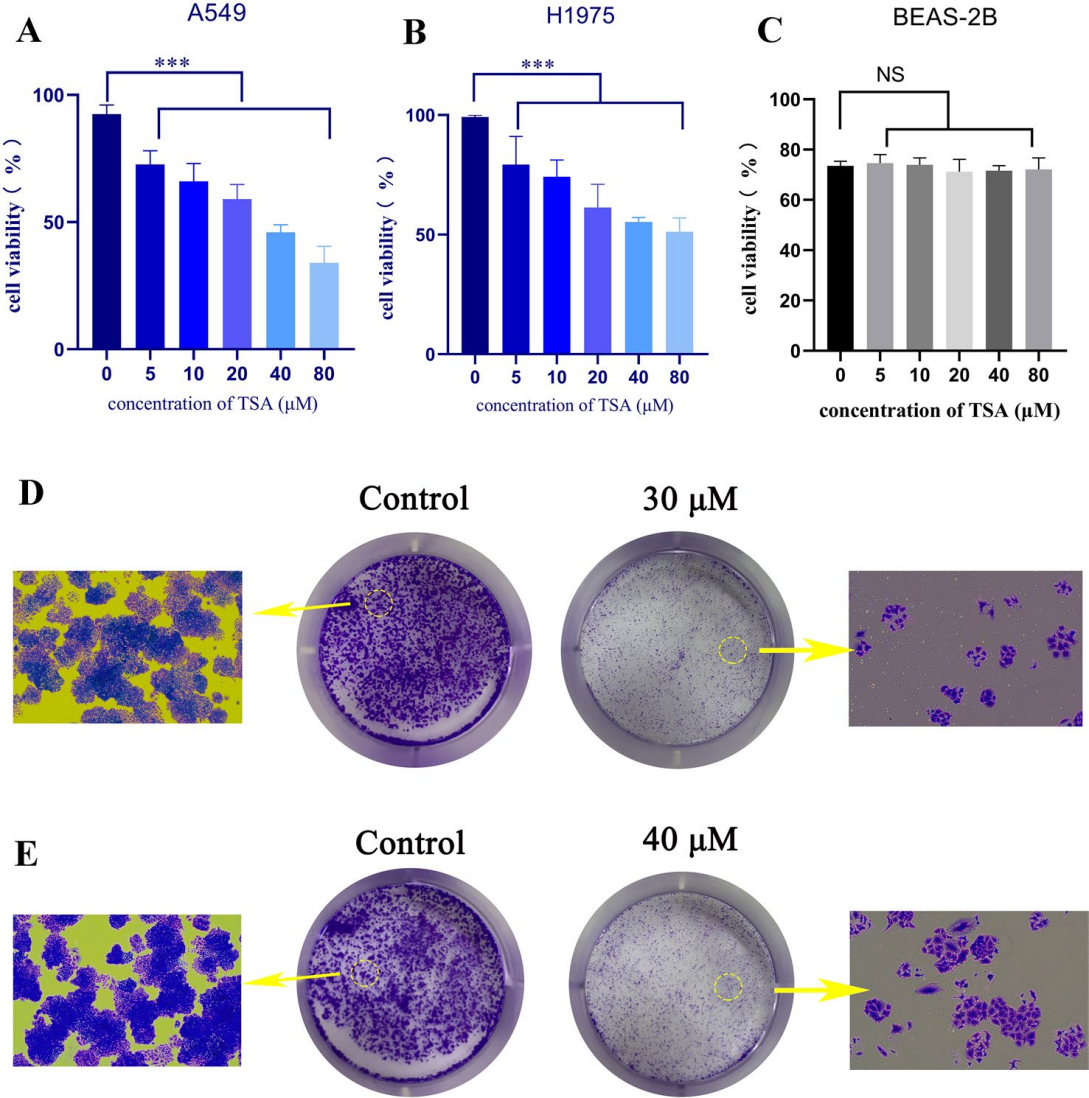
Effect of TSA on the viability of A549 (**A**), NCI-H1975 (**B**) and BEAS-2B (**C**) cells, after treatment with TSA for 24 h, the viability of these cells was measured by MTT assay, ***P < 0.001 vs. control. Plate clone formation assay, the A549 (**D**) cells were cultured with TSA (0, 30 µM) and H1975 (E) were cultured with TSA (0, 40 µM) 14 days.

Human lung adenocarcinoma A549 and NCI-H1975 cells were used to determine the anticancer activity of TSA. MTT assay results showed that with the increase of the concentration, the inhibition ability of TSA on the two kinds of cells gradually increased (*P* < 0.01). The half-maximal inhibitory concentration (IC50) of TSA for A549 was about 30 μM, and for NCI-H1975 was about 40 μM (Fig. 6A,B). Normal human lung epithelial cells BEAS-2B were found to be insensitive to TSA (Fig. 6C). The above-mentioned results suggested that TSA exhibited a good selectivity between LUAD cells and normal lung cells. The plate cloning assays also verified that TSA significantly inhibited the proliferation of LUAD cells (A549, Fig. 6D; and NCI-H1975, Fig. 6E).

### TSA induces apoptosis and suppresses G1/S transition in LUAD cells

**Figure 7:**
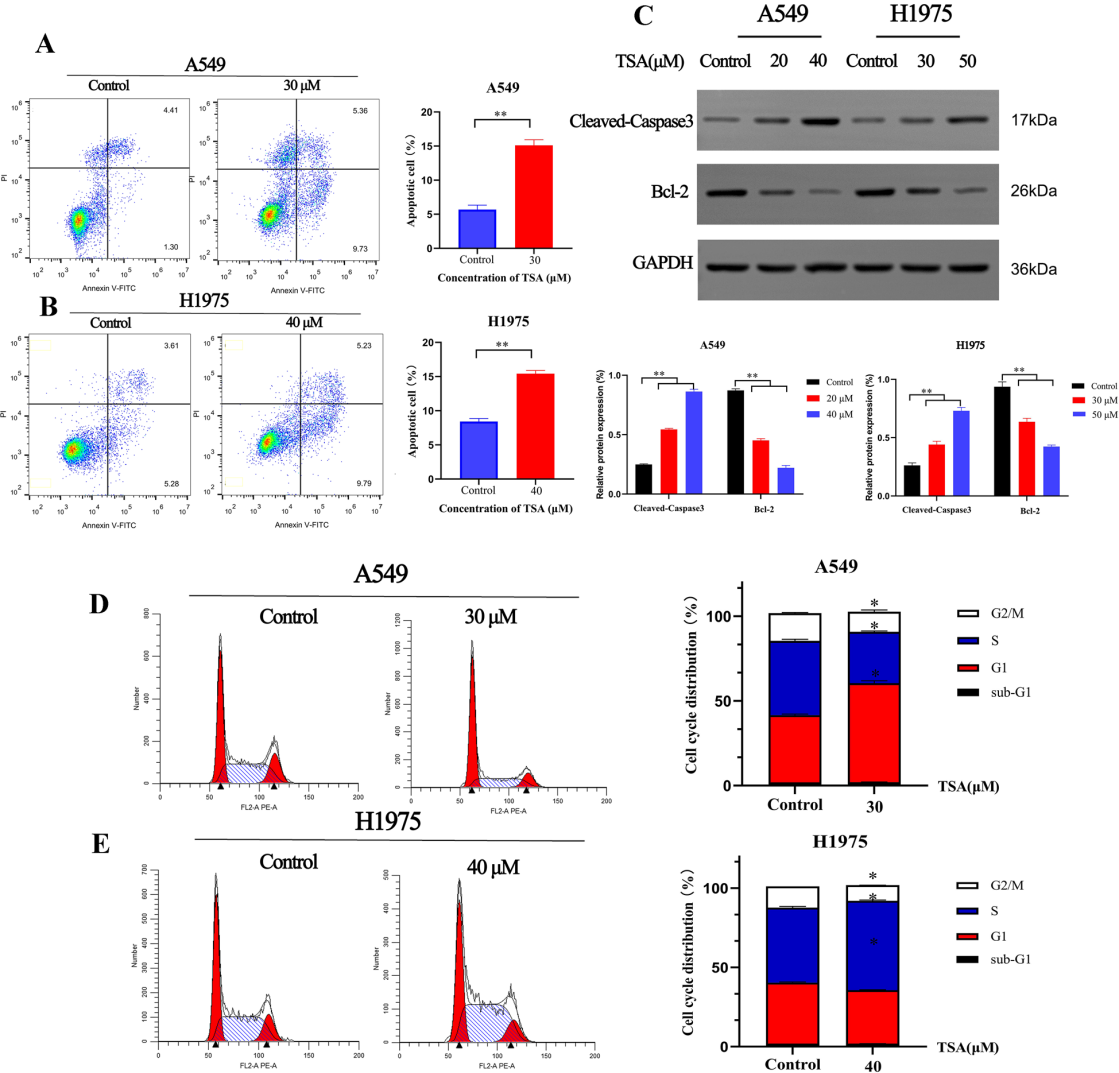
Apoptosis induction and cell cycle arrestion of A549 and H1975 after treating with TSA. The apoptosis of A549 (**A**) and H1975 (**B**) was evaluated by flow cytometric analysis after staining for PI and Annexin V. (**C**) The expression level of apoptotic marker protein was detected by western blot assay. **P < 0.01 vs. control. A549 (**D**) and H1975 (**E**) cells were infected with TSA, and cell cycle progression was analyzed by flow cytometry. *P < 0.05 vs. control.

To explore the apoptosis induction effect of TSA on LUAD cells, flow cytometry analysis and Western Blot assay were performed. As shown in Fig. 7A,B, compared with control group, the apoptosis markedly increased in the treated with TSA in both cell lines, but A549 had more apoptosis than H1975. To confirm that apoptosis did occurred in TSA-treated cells, we measured the expression of apoptosis-related signature proteins using western blot assay. As shown in Fig. 7C, a significant increased expression of Cleaved-Caspase3 was observed in TSA groups when compared with the control group (*P* < 0.01). And compare with the control group, the expression of Bcl-2 was markedly decreased (*P* < 0.01). When the two groups of cell lines were compared, the regulation effect of TSA on apoptosis-related proteins in A549 cells was more obvious than that in H1975 cells. Further, flow cytometry was used to analyze the cell cycle phases of the TSA treated cells. As shown in Fig. 7D,E, TSA arrested the A549 at G1 phases and H1975 at S phase. These findings collectively proposed that TSA could induce the apoptosis of LUAD cells, and suppress G1/S transition which may be the important reasons for its inhibition of lung cancer.

### TSA mediates oncogenic effects on LUAD cells via inhibiting CCNA2-CDK2 complex and PLK1/ERK pathway

**Figure 8:**
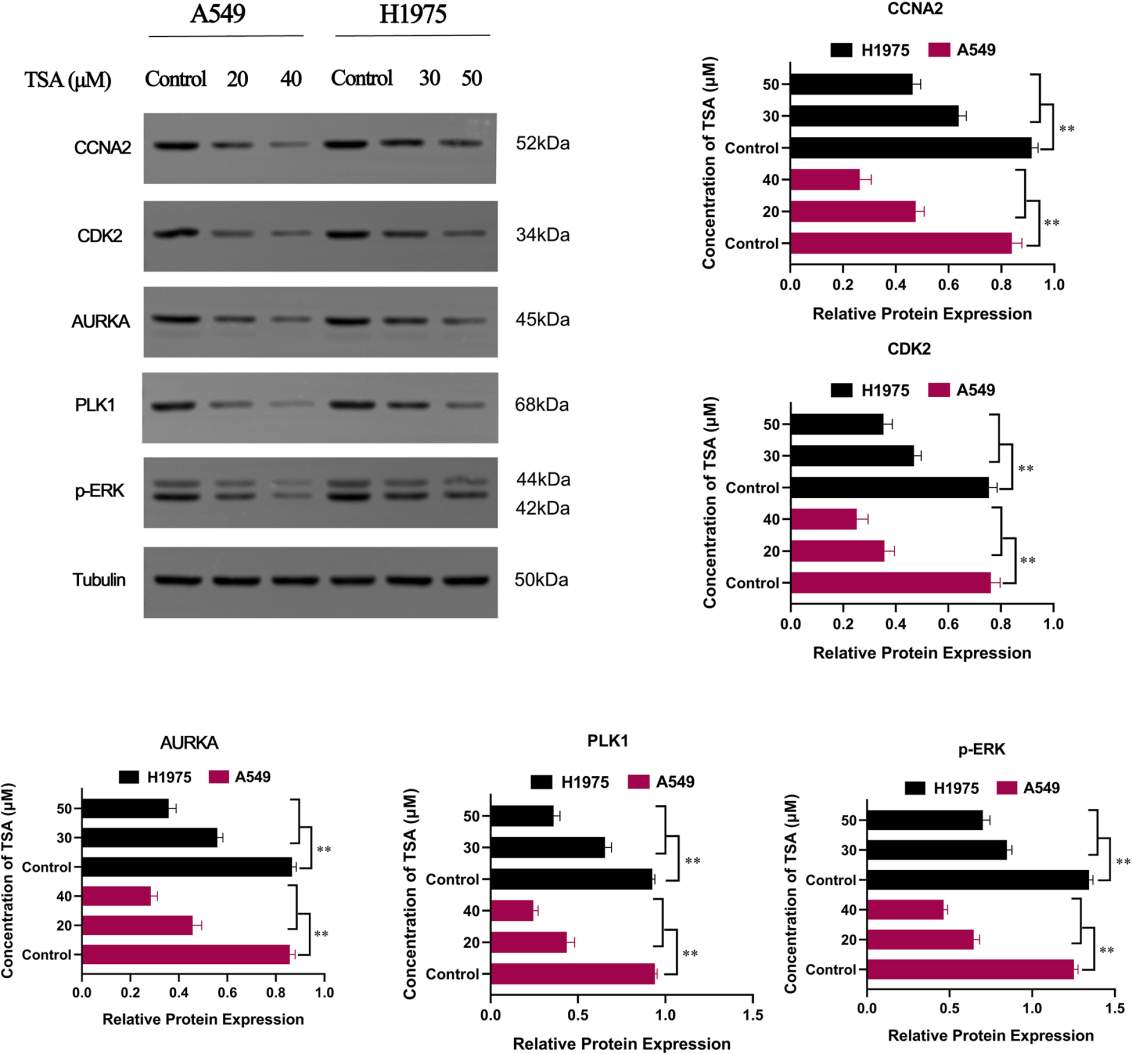
Expressions of CCNA2, CDK2, AURKA, PLK1, and p-ERK were detected using western blot assay. Quantitation of the western blot analysis for CCNA2, CDK2, AURKA, PLK1, and p-ERK compared with GAPDH, and **P < 0.01 versus the control group.

We then inspected the molecular mechanisms of relevant genes screened by bio-informatics analysis and molecular docking via western blot assay. CCNA2-CDK2 complexes were generally considered to be key regulatory proteins in the G1 and S phases. Our study demonstrated that TSA could downregulate CCNA2 and CDK2 in a dose-dependent manner in LUAD cells (*P* < 0.01) (Fig. 8). AURKA, PLK1 and ERK played a critical role in chromosomal instability, cell cycle progression, and maturation. The Western Blot assay results shown that TSA downregulated the expression of these three proteins (*P* < 0.01).

## Materials and methods

### Data collection

TCGA (https://portal.gdc.cancer.) is a database built by American National Cancer Institute(NCI) and NHGRI contains information on 36 types of cancer. We downloaded the data of 1014 cases of LUAD patients and 686 cases of normal tissues from TCGA. PubChem (https://pubchem.ncbi.nlm.nih.gov/) is a database of chemical modules. The canonical SMILES and 2D structure of TSA were contained from the PubChem. The target genes associated with TSA were retrieved from the SwissTargetPrediction (http://www.swisstargetprediction.ch/) database which allowed people to estimate the most probable macromolecular targets of a small molecule, assumed as bioactive. Because TCGA's data are public, the study was exempt from approval by a local ethics committee.

### Identification of DEGs

Differential expression was assessed through R statistical package software EdgeR (Empirical analysis of Digital Gene Expression in R). Genes with adjusted *P* < 0.05 and |log2 fold change (FC)|> 1.0 were considered to be DEGs in the combined analysis. Then, Venny2.1.0 database was used to obtain intersection of the DEGs and TSA-related genes, and these genes were looked as the target genes of TSA for treatment of LUAD.

### Protein–protein interaction network construction

SRTING is a database of known and predicted protein–protein interactions developed by European Molecular Biology Laboratory. The interactions include direct (physical) and indirect (functional) associations, they stem from computational prediction, from knowledge transfer between organisms, and from interactions aggregated from other (primary) databases. Target genes were put into STRING to build a protein–protein interactions network.

### GO and KEGG pathway enrichment analysis

We put the target genes into the Database for Annotation, Visualization, and Integrated Discovery (DAVID, https://david.ncifcrf.gov/) to process data and visualize the enrichment results of gene ontology (GO) and Kyoto Encyclopedia of Genes and Genomes (KEGG) pathway enrichments^14^. Then we put the top ten pathways and their associated genes into Cytoscape 3.7.2 (http://www.cytoscape. org/) to reflect the complex relationships between pathways and their related genes. In addition, the results obtained were visualized by Omicshare (https://www.omicshare.com/).

### GEPIA database-based DEGs confirmation and overall survival analysis

GEPIA (http://gepia.cancer-pku.cn/index.html) contains sequence expression data of 9736 tumors and 8587 normal tissues from TCGA and GTEX projects. GEPIA provides customizable functions such as tumor/normal differential expression analysis, profiling according to cancer types or pathological stages, patient survival analysis, similar gene detection, correlation analysis and dimensionality reduction analysis. In this study, " Expression on Box Plots" module was used to perform differential expression analysis of target genes. " Survival Plots" module was used to perform differential overall survival analysis of target genes. " Correlation Analysis" module was used to further verify the interrelationships of genes.

### Molecular docking

The ".sdf" format structure of TSA was downloaded from PubChem. The structure of proteins was downloaded from RSCB PDB database (https://www.rcsb.org/). The structures of compound and proteins were imported into AutoDockTools-1.5.6 software for calculating the total charge. High-quality 3D structures of compound and proteins were created and the protein residues and binding bonds were displayed via PyMol, a software for pre-docking small molecule components and proteins. We considered the binding effective when the binding energy was below -4 kcal/mol^15^.

### Cell culture

The human lung cancer cell lines A549 and NCI-H1975 were obtained from Cell Bank of the Chinese Academy of Sciences (Shanghai, China). Human lung epithelial cells BEAS-2B and mouse Lewis lung carcinoma cells (LLC) were purchased from Procell Life Science & Technology Co., Ltd. (Wuhan, China). A549 were complemented in Ham’s F-12 K (Grand Island, NY) supplemented with 10% fetal bovine serum (FBS, Gibco, USA) and 1% P/S solution (Gibco, MD, USA). NCI-H1975, BEAS-2B and LLC were complemented in DMEM medium containing (Grand Island, NY) with 10% fetal bovine serum and 1% P/S solution. Cell lines were cultured at 37 °C in 5% CO2.

### Cell proliferation detection

MTT assays were carried out to detect the effect of TSA on A549 cell, NCI-H1975 and BEAS-2B cell activity. TSA was purchased from Shanghai Yuanye Biotechnology Co., Ltd (Shanghai, China.). A549 and NCI-H1975 cell lines were seeded into 96-well plates at density of 5000 cells/well and cultured 12 h at 37℃. Then, cells were treated with TSA in different concentrations (0, 5, 10, 20, 40, 80 µM) for 24 h. After that, MTT solution (Sigma, USA) was added 200 µL each well, and the cells were incubated at 37 °C for 4 h. 150 µL dimethyl sulfoxide (DMSO) (Sigma, USA) was added to every well. The absorbance at 490 nm was measured on Spark 10 M microplate reader (Tecan, Männedorf, Switzerland).

### Plate cloning experiment

A549 and NCI-H1975 cells were inoculated into 6-well plates at 500 cells/well. Cells were treated with different concentrations of TSA (A549, 0, 30 µM; NCI-H1975, 0, 40 µM), respectively. After culturing for 14 days, cells were fixed with polyoxymethylene for 10 min, washed 3 times with PBS, stained with Giemsa stain solution for 15 min. The colony formation was observed and photographed under a microscope.

### Annexin V-FITC and PI dual staining assay

To test the effect of TSA on the apoptosis of lung adenocarcinoma cells, A549 cells were plated into 6-well plates with TSA (0, 30 µM) for 48 h, and NCI-H1975 were treated with TSA (0, 40 µM). Then cells were trypsinized and washed with PBS twice, suspended in 200 ΜL binding buffer. After that, cells were staining with FITC Annexin V and 10 μL PI (Beyotime Biotechnology, Shanghai, China) for 30 min at 4 ℃. The stained cells were analyzed in the FL2 channel of a flow cytometer BD FACSCalibur (BD Bioscience, Franklin Lakes, NJ, USA) with a 488 nm excitation laser. Data were analyzed using FlowJo (FlowJo, LLC, Ashland, OR, USA).

### Cell cycle analysis

Same as apoptosis analysis, A549 and NCI-H1975 cells were seeded into 6-well plates at density of 1 × 105 cells / well for 24 h. After treated with various concentrations of TSA (A549, 0, 30 µM; NCI-H1975, 0, 40 µM) for 24 h. After discarding the medium, cells were washed twice with PBS, fixed with 70% ethanol for 2 h. The ethanol was removed by centrifugation, cells were washed twice with PBS, and 0.5 mL propidium iodide (PI, Sigma, USA) staining solution was added for 30 min in dark at 37 ℃. Flow cytometer BD FACSCalibur was used to analyzed distribution of DNA content. Data were analyzed using FlowJo.

### Western blot analysis

Same as before with cell culture, the cell protein was extracted using RIPA lysis buffer (Beyotime Institute of Biotechnology, China). BCA protein assay kit (AS1086, ASPEN, Wuhan, China) was used to achieve total protein quantification. Samples were separated by SDS PAGE and electro-transferred onto the NC membrane (Millipore, USA). They were then incubated overnight at 4 °C with the specific primary antibodies (CCNA1, CDK1, AURKA, PLK1, p-ERK, Cleaved-caspase3, Bcl-2, Tubulin, GAPDH, Cell Signaling Technology, MA, USA). The membranes were washed three times with TBST for 10 min. After washing, the membranes were incubated with HRP anti-conjugated secondary antibodies (Cell Signaling Technology, MA, USA) for 1 h. The membranes were washed in the same manner as described above. The membranes were scanned using the Fluor Chem FC3 system (Protein Simple, USA).

### Statistical analysis

In this study, ANOVA was used to analyze the expression of target genes in GEPIA database. |Log2fc| cutoff < 1 and Q-value < 0.05 were considered to be significant. Overall survival (OS) was analyzed based on the Mantel–Cox test and Log rank *P* < 0.01 were considered to be significant. Significant data in GO and KEGG pathway enrichment were screened according to *P* < 0.05 with students' t-test. Relevant data of in vitro experiment were performed using SPSS 22.0 software (IBM, USA). Data were expressed as the mean ± SD. All experiments were repeated in triplicate and *P* < 0.05 was considered significant.

## Discussion

Recent years, with the progress of genome research and the development of modern biotechnology, a large amount of biological data has been accumulated, which leads to the unprecedented development of bio-information analysis^16^. More and more natural product pharmacology is looking for researching direction from bioinformatics and network pharmacology. Many researchers have expanded genomics and bioinformatics in Traditional Chinese Medicine (TCM) theory, explained the molecular mechanism of TCM clinical efficacy, deepened the research on important genes, and further developed the molecular pharmacology of TCM^17^. Zhang et al. found that active ingredients of *Rheum palmatum* L. had anti-lung cancer effect via induction of apoptosis by applying network pharmacology method^18^. Hong et al. used bio-information analysis method to found gallic acid can inhibit the growth of colon cancer cells, and its mechanism of action is related to ferroptosis^19^. In this study, we used a combination of bio-information analysis and network pharmacology, as well as molecular docking, to simulate the possible mechanisms of TSA treatment for LUAD.

At the beginning of the study, we predicted the DEGs associated with TSA in LUAD and obtained 10 key genes that affect the overall survival of patients. Then the results of molecular docking showed that among the ten genes, CCNA2-CDK2 complex showed the strongest binding to TSA. The Cell cycle is a continuous, tightly controlled process that is regulated by three types of proteins: cyclins, cyclin-dependent kinases (CDKs), and cyclin-dependent kinase inhibitors (CKIs)^20^. Abnormalities in the proteins involved in the cell cycle often lead to tumorigenesis^21^. The Cyclin protein family has the same structural characteristics. They all contain a sequence of amino acids called the Cyclin box, which is about 100 amino acids long^22^. This box’s function is to bind to the CDKs, thus exerting enzyme activity. In general, different cyclins bind to different CDKs to form complexes that function at different stages of the cell cycle, thus promoting the normal cell cycle. Cyclin A2 (CCNA2) has been found to commonly bind to CDK2 to form complexes that regulate the G1 to S phase or the S to G2 phase^23^. Studies have shown that CCNA2 is often overexpressed in lung cancer, colorectal cancer, and liver cancer^24^. Currently, inhibitors of CCNA-CDK complex have been considered to be an important target for the treatment of cancer.

In this study, we conducted a variety of biological experiments to verify whether the mechanism of TSA in treating LUAD is achieved through inhibition of CCNA2-CDK2. At first, flow cytometry results proved that TSA could indeed induce cycle arrest in LUAD cells, although A549 and H1975 induced cycle arrest were in the G1 phase and the S phase, respectively, this result was consistent with the results shown in many studies. Secondly, from western blot results, we know that the expression of both CCNA2 and CDK2 were down-regulated, which further confirmed our experimental hypothesis. However, the problem of our study is that we did not further investigate the site of TSA inhibition on CCNA2-CDK2 complex, which is what we need to focus on in the following work.

Based on the results of bioinformatics analysis and molecular docking, we also found that aurora kinase A (AURKA) and Polo-like kinase 1 (PLK1) may also be the key proteins regulating TSA anti-LUAD. PLK1 is known to play an important role in initiating, completing, and maintaining the cell mitotic cycle^25^. Elevated levels of PLK1 transcription, or protein expression, have been found in a variety of human solid tumors, including lung, breast, stomach, cervical, and colon cancers^26^. Multiple studies have shown that PLK1/ERK is a key pathway to induce malignant transformation and tumor formation of cancer cells. Gao et al. found that PLK1 could promotes proliferation and suppresses apoptosis of renal cell carcinoma cells by phosphorylating MCM3^27^, which suggested that PLK1 was also closely related to cell cycle and apoptosis. N. Tavernier et al. found that CCNA2-CDK2 complex could activate Bora phosphorylation, and p-Bora bind to AURKA and PLK1 to form a complex that activates PLK1 expression, thus promote cell proliferation, inhibit cell apoptosis^28^.

But at present, there are still some deficiencies in our research. More direct evidence needs to be provided that TSA does indeed treat lung adenocarcinoma through the CCNA2-CDK2 complex and AURKA/PLK1 molecule. We designed a more feasible experiment to verify our idea. We treated A549 and 1975 with TSA, NU5300 (an inhibitor of CDK2)^29^, and a combination of TSA to observe their effects on the reproduction of LUAD cells. The results are shown in Supplementary Fig. 1. From the results, we know that the combination of NU6300 and TSA had superimposed reproductive inhibition on LUAD cells, this suggests that TSA and NU6300 target the same protein, CDK2. It is not hard to argue that more accurate experiments should be carried out, such as surface plasmon resonance (SPR) or microscale thermophoresis (MST), but given the time and cost of the trial, we can only prove it by simple and feasible experiments of the present kind. Our research group will study this part in detail in the next stage.

To verify whether TSA promotes apoptosis of LUAD cells through CCNA2-CDK2 and AURKA/PLK1/ERK pathways, we performed a series of experiments in vitro. The results of Annexin V-FITC and PI dual staining assay showed that different concentrations of TSA could promote the apoptosis of LUAD cells, the results of western blot demonstrated that TSA down-regulated the expression of AURKA, PLK1, and p-ERK. These findings all proved that TSA inhibited the proliferation of LUAD cells by inducing cell cycle arrest and apoptosis through CCNA2-CDK2 and AURKA/PLK1 /ERK pathways.

## Conclusion

This study is the first to combine bio-information analysis with experimental validation to investigate the mechanism of TSA against LUAD. Taken together, TSA was verified to suppress the progression of lung adenocarcinoma by inducing cell apoptosis, arresting cell cycle through regulating CCNA2-CDK2 complex and AURKA/PLK1 pathway. This study provides a theoretical basis for the clinical treatment of LUAD using TSA, but there are some limitations at the same time. More studies in vitro and in vivo are needed to verify our results. We will explore this in more depth in the next stage.

## Supplementary Information

Supplementary Information.

## Data Availability

The datasets used and/or analysed during the current study were available from the corresponding author on reasonable request. The main supporting data can be found in the supplementary material of the article.

## References

[1] Rodriguez-Canales J, Parra-Cuentas E, Wistuba II (2016). Diagnosis and molecular classification of lung cancer. Cancer Treat. Res..

[2] Bray F (2018). Global cancer statistics 2018: GLOBOCAN estimates of incidence and mortality worldwide for 36 cancers in 185 countries. CA Cancer J. Clin..

[3] Bade BC, Dela Cruz CS, Cancer L (2020). Epidemiology, etiology, and prevention. Clin. Chest. Med..

[4] Pascoe HM, Knipe HC, Pascoe D, Heinze SB (2018). The many faces of lung adenocarcinoma: a pictorial essay. J. Med. Imaging Radiat. Oncol..

[5] Steffen McLouth LE (2020). Patient-reported outcomes from patients receiving immunotherapy or chemoimmunotherapy for metastatic non-small-cell lung cancer in clinical practice. Clin. Lung Cancer.

[6] Duan J (2020). Refined stratification based on baseline concomitant mutations and longitudinal circulating tumor DNA monitoring in advanced EGFR-mutant lung adenocarcinoma under gefitinib treatment. J. Thorac. Oncol..

[7] Takemoto H (2020). Polymeric modification of gemcitabine via cyclic acetal linkage for enhanced anticancer potency with negligible side effects. Biomaterials.

[8] Malumbres M (2014). Cyclin-dependent kinases. Genome Biol..

[9] Wu J (2014). MicroRNA-188 suppresses G1/S transition by targeting multiple cyclin/CDK complexes. Cell Commun. Signal.

[10] Casado-Vela J, Martinez-Torrecuadrada JL, Casal JI (2009). Differential phosphorylation patterns between the Cyclin-A2/CDK2 complex and their monomers. Protein Expr. Purif..

[11] Zhou ZY, Zhao WR, Zhang J, Chen XL, Tang JY (2019). Sodium tanshinone IIA sulfonate: a review of pharmacological activity and pharmacokinetics. Biomed. Pharmacother..

[12] Liu L (2021). Tanshinone IIA attenuates AOM/DSS-induced colorectal tumorigenesis in mice via inhibition of intestinal inflammation. Pharm. Biol..

[13] Wang Y, Jin W, Wang J (2021). Tanshinone IIA regulates microRNA125b/foxp3/caspase1 signaling and inhibits cell viability of nasopharyngeal carcinoma. Mol. Med. Rep..

[14] Kanehisa FM, Tanabe M, Sato Y, Morishima K (2017). KEGG: new perspectives on genomes, pathways, diseases and drugs. Nucleic Acids Res..

[15] Trott O, Olson AJ (2010). AutoDock Vina: improving the speed and accuracy of docking with a new scoring function, efficient optimization, and multithreading. J. Comput. Chem..

[16] Hua P, Zhang Y, Jin C, Zhang G, Wang B (2020). Integration of gene profile to explore the hub genes of lung adenocarcinoma: a quasi-experimental study. Medicine (Baltimore).

[17] Li S, Zhang B (2013). Traditional Chinese medicine network pharmacology: theory, methodology and application. Chin. J. Nat. Med..

[18] Zhang Q (2020). A network pharmacology approach to investigate the anticancer mechanism and potential active ingredients of *Rheum palmatum* L. against lung cancer via induction of apoptosis. Front. Pharmacol..

[19] Hong Z (2021). Ferroptosis-related genes for overall survival prediction in patients with colorectal cancer can be inhibited by gallic acid. Int. J. Biol. Sci..

[20] Lim S, Kaldis P (2013). Cdks, cyclins and CKIs: roles beyond cell cycle regulation. Development.

[21] Kastan MB, Bartek J (2004). Cell-cycle checkpoints and cancer. Nature.

[22] Anand K, Schulte A, Fujinaga K, Scheffzek K, Geyer M (2007). Cyclin box structure of the P-TEFb subunit cyclin T1 derived from a fusion complex with EIAV tat. J. Mol. Biol..

[23] Hein JB, Nilsson J (2016). Interphase APC/C-Cdc20 inhibition by cyclin A2-Cdk2 ensures efficient mitotic entry. Nat. Commun..

[24] Li JA, Liu BC, Song Y, Chen X (2018). Cyclin A2 regulates symmetrical mitotic spindle formation and centrosome amplification in human colon cancer cells. Am. J. Transl. Res..

[25] Zhang C (2021). STK39 is a novel kinase contributing to the progression of hepatocellular carcinoma by the PLK1/ERK signaling pathway. Theranostics.

[26] Ocklenburg T (2021). In oxygen-deprived tumor cells ERp57 provides radioprotection and ensures proliferation via c-Myc, PLK1 and the AKT pathway. Sci. Rep..

[27] Gao Z (2020). PLK1 promotes proliferation and suppresses apoptosis of renal cell carcinoma cells by phosphorylating MCM3. Cancer Gene. Ther..

[28] Tavernier N (2021). Bora phosphorylation substitutes in trans for T-loop phosphorylation in Aurora A to promote mitotic entry. Nat. Commun..

[29] Anscombe E, Meschini E, Mora-Vidal R (2015). Identification and Characterization of an Irreversible Inhibitor of CDK2. Chem. Biol..

