# Supplementary material for: Tanshinone IIA suppresses the progression of lung adenocarcinoma through regulating CCNA2-CDK2 complex and AURKA/PLK1 pathway
Source: Sci Rep. 2021 Dec 8;11:23681. doi: 10.1038/s41598-021-03166-2 (PMC8654884; doi:10.1038/s41598-021-03166-2)

**Tanshinone ⅡA suppresses the progression of lung adenocarcinoma by inducing cell apoptosis, arresting cell cycle through regulating CCNA2-CDK2 complex and AURKA/PLK1 pathway**

Ziheng Li^1#^, Ying Zhang^1#^, Yuan Zhou^1^, Fuqian Wang^1^, Chao Yin^1^, Li Ding^1*^ and Shunbo Zhang^1*^

1.Faculty of Pharmacy, Hubei University of Chinese Medicine, Wuhan 430065, China

* Correspondence:

Li Ding, email: 17767153@qq.com

Shun-Bo Zhang, email: zshunbo163@163.com

# Ziheng Li and Ying Zhang contributed equally to this work.

Supplementary Table S1. 64 DEGs related to TSA

| Gene official symbol | | | |
| --- | --- | --- | --- |
| MIF | AURKA | MMP12 | PARP1 |
| SORD | CRABP2 | CCNA2 | CASP3 |
| PLK1 | HADH | MTHFD1 | GLO1 |
| MET | ADK | HNF4G | DCK |
| GM2A | GSR | MMP13 | MMP7 |
| NQO1 | CBR1 | GSTA1 | LCN2 |
| AKR1C1 | ADAM33 | PADI4 | CFD |
| F10 | FABP4 | PGR | ELANE |
| IGF1 | TNNC1 | ARG1 | PDE5A |
| HSD11B1 | FGFR1 | MAOB | ACE |
| LTA4H | RARA | PNMT | THRA |
| BTK | PDE3B | CASP1 | KDR |
| JAK2 | PLA2G2A | WAS | MMP8 |
| JAK3 | PPARG | HPGDS | ANG |
| NOS3 | RBP4 | BCHE | HCK |
| BMP2 | ALB | SULT1E1 | CES1 |


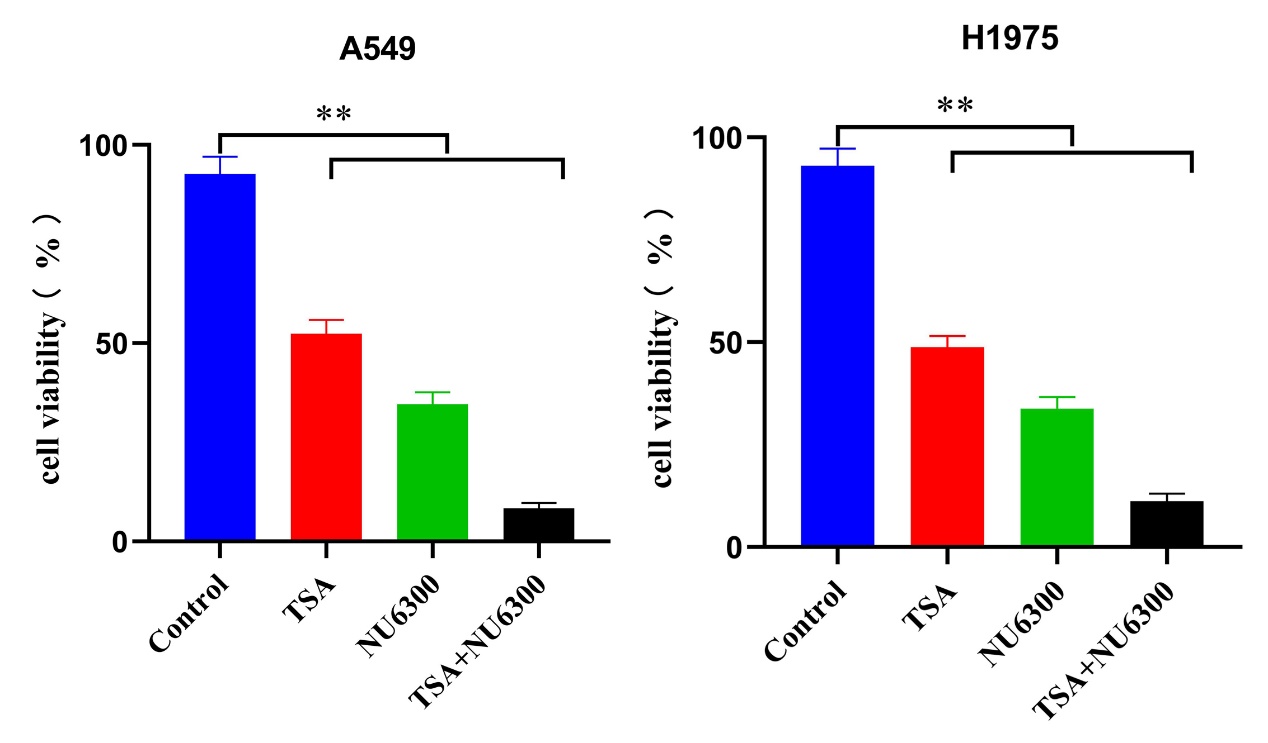


**Supplementary Figure 1** Effect of TSA and NU6300 on the viability of A549 and NCI-H1975 cells.

**Supplementary information about blots**

**Figure7**


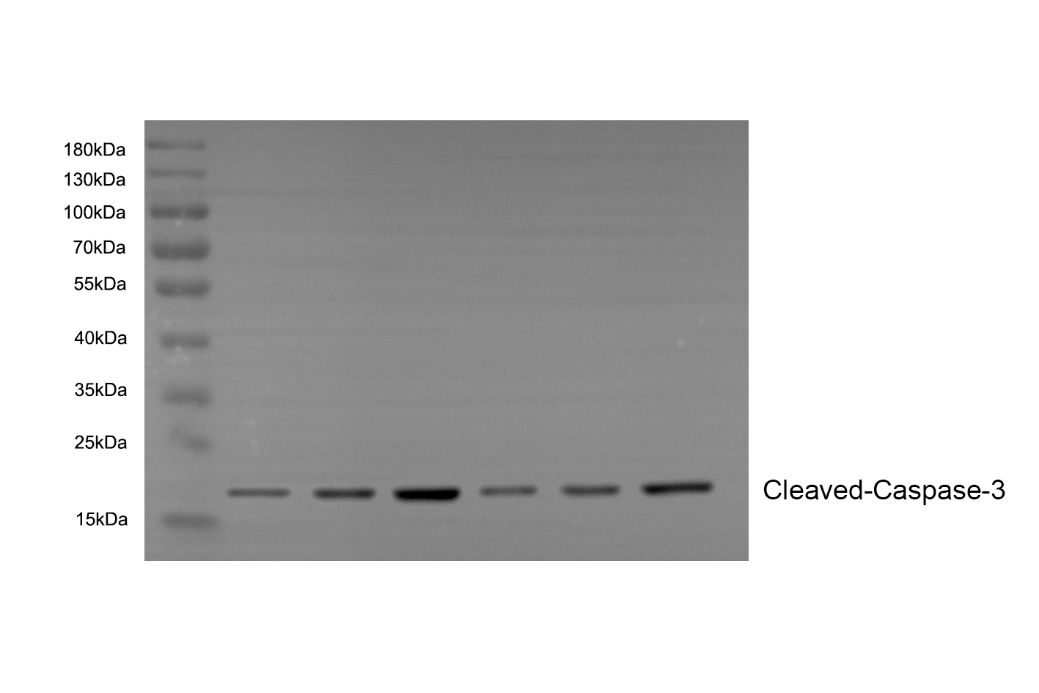


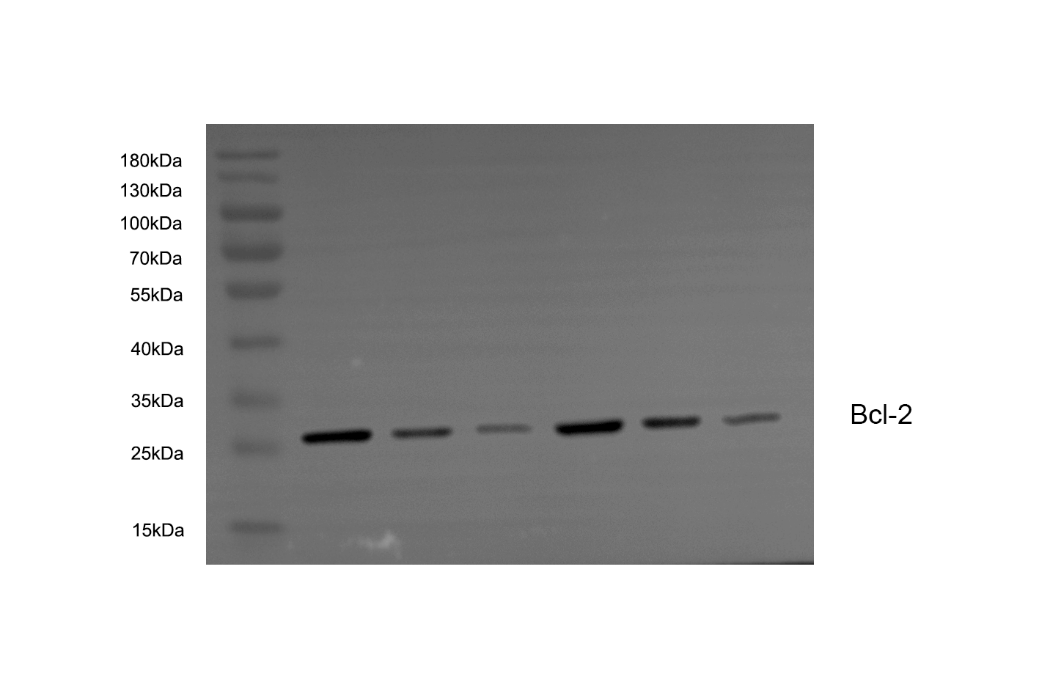


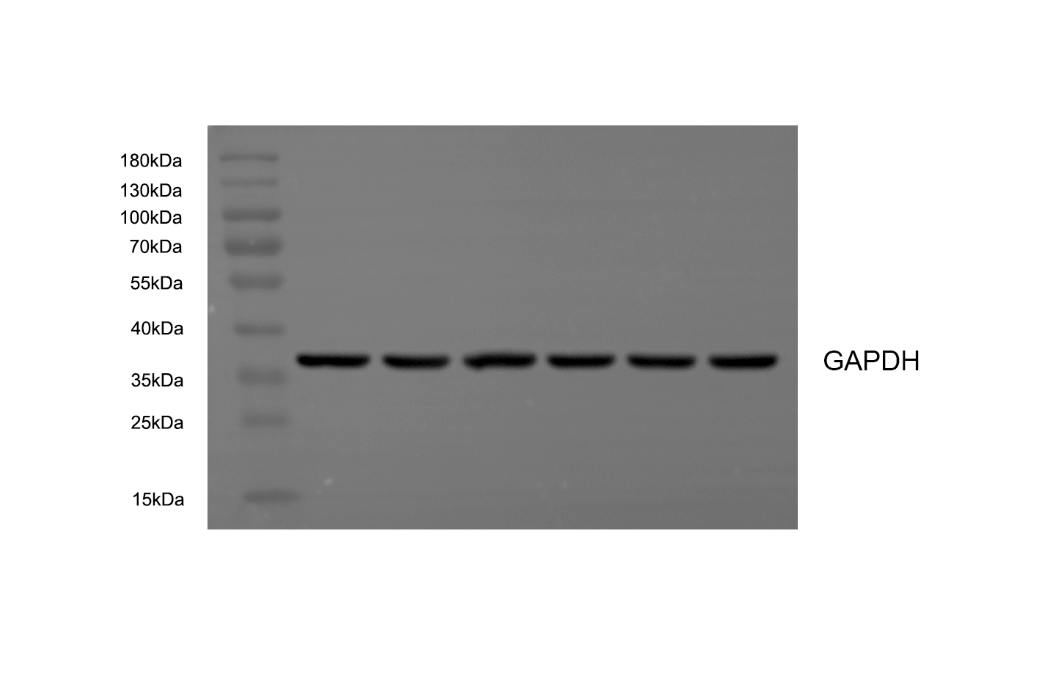


**
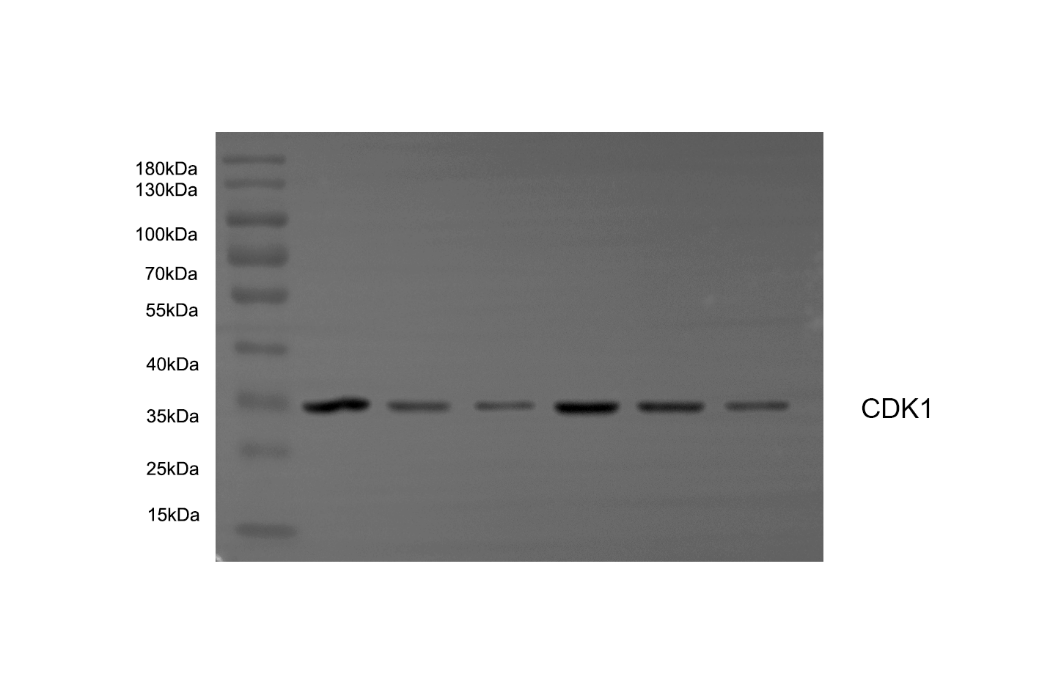

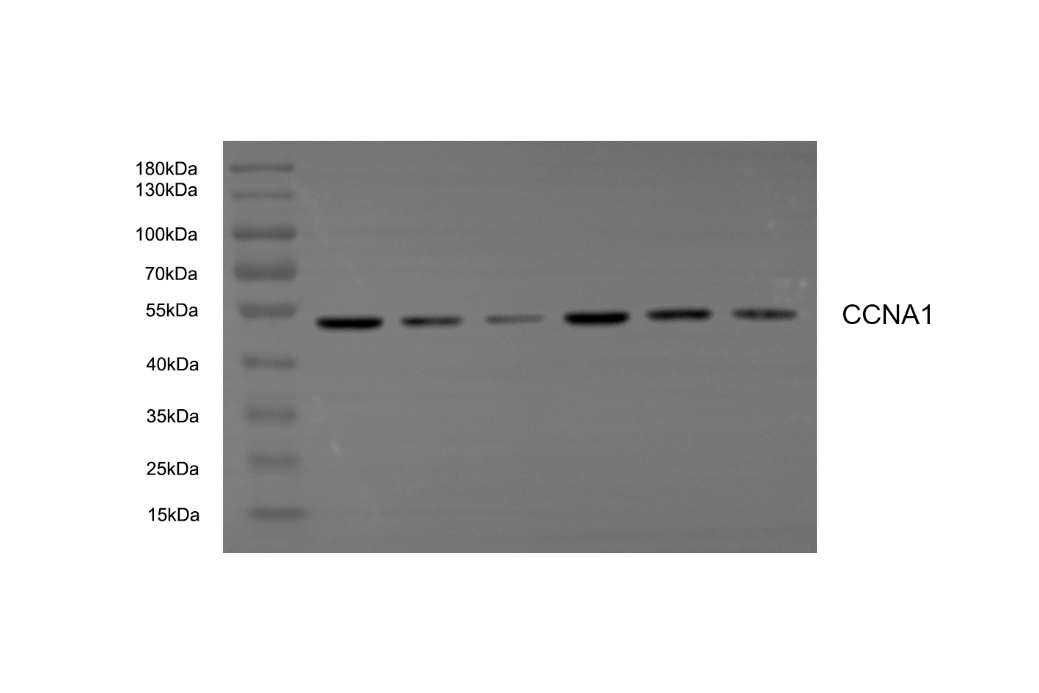
Figure8**


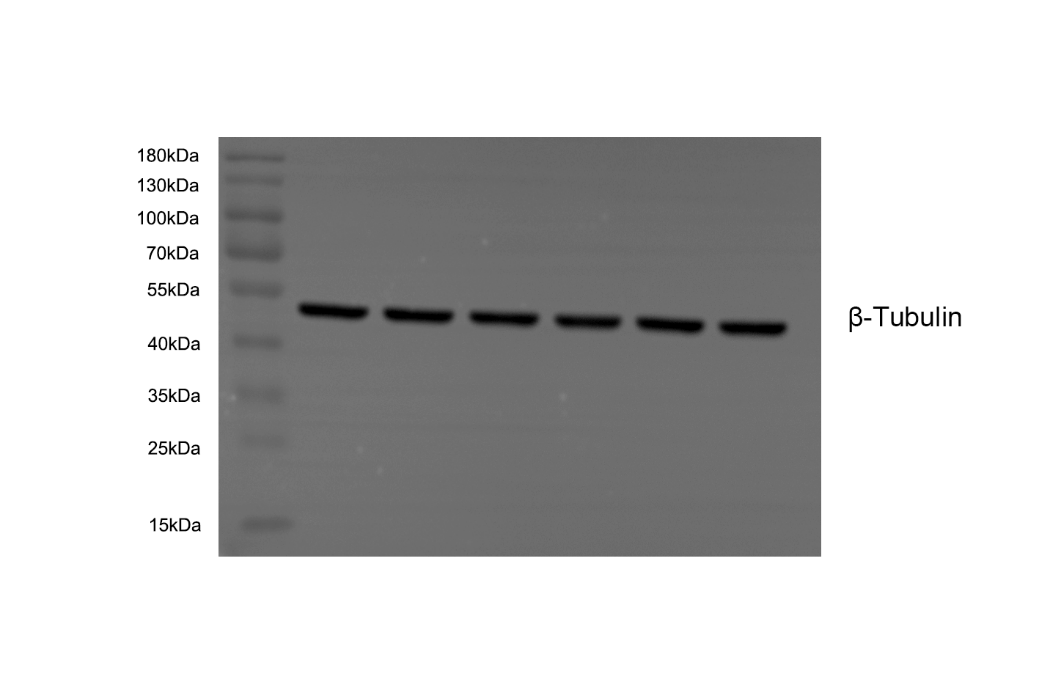

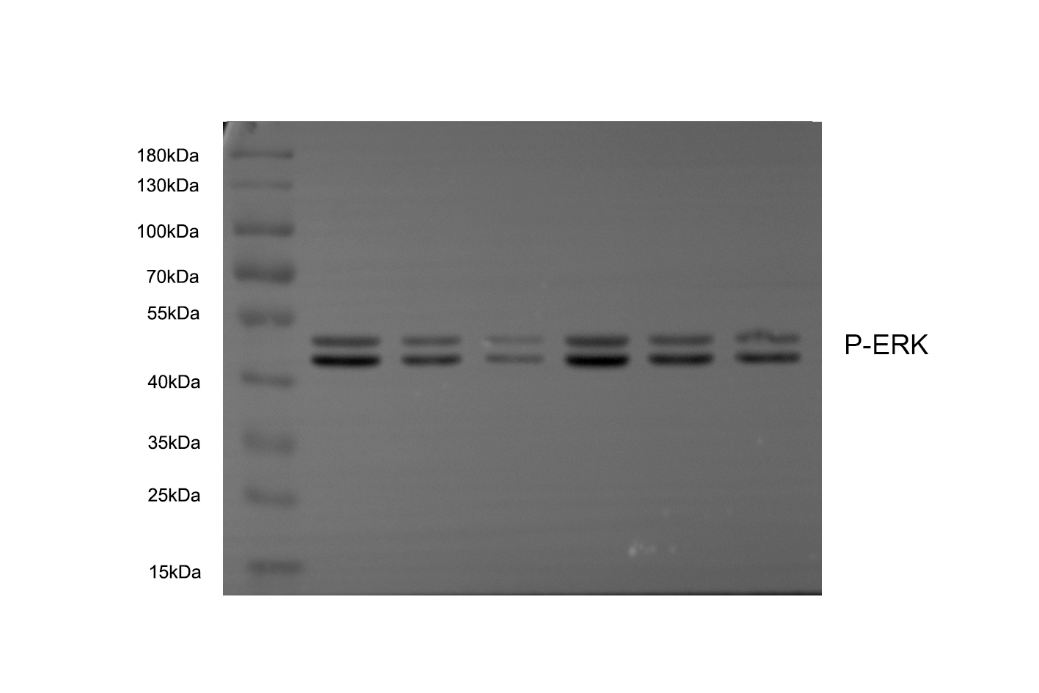

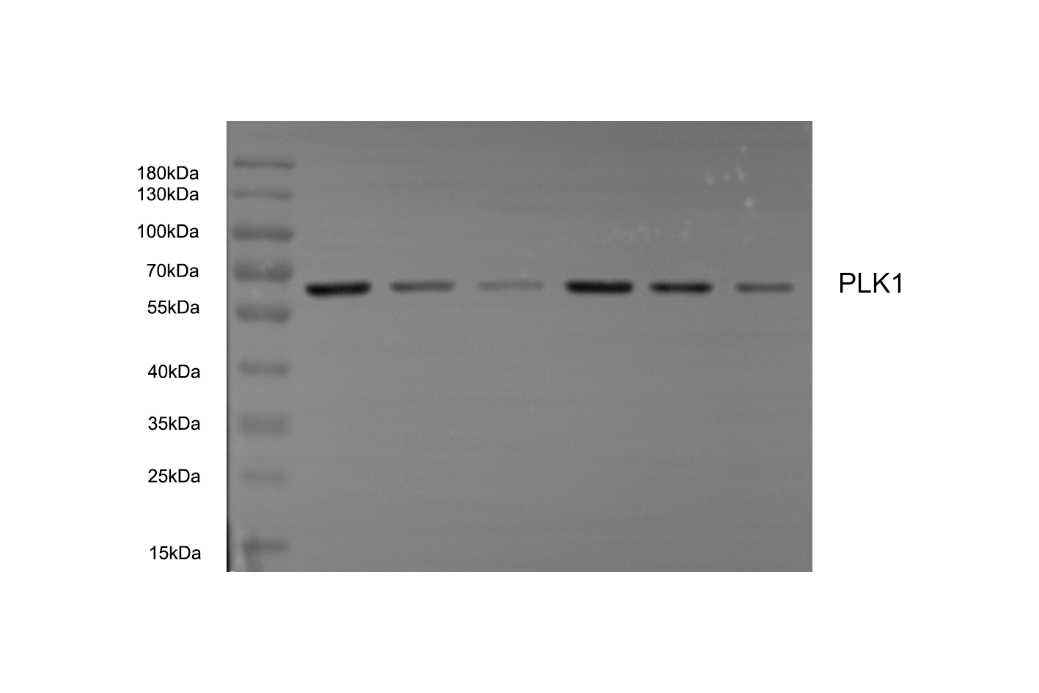

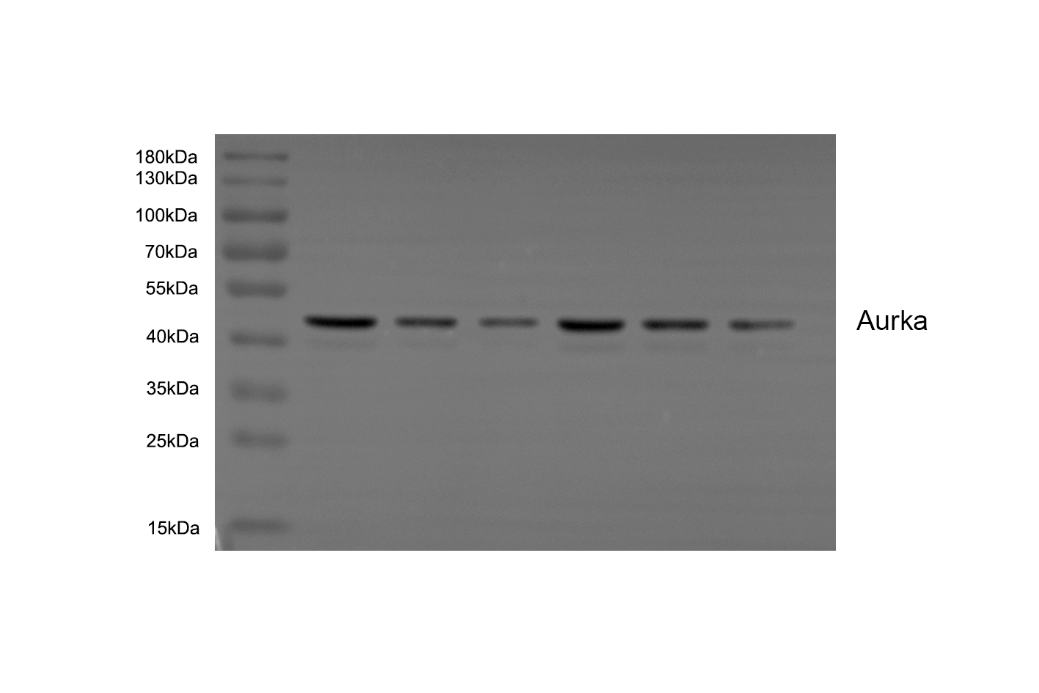

Supplement: Supplementary file 1 — Supplementary Information. [file 41598_2021_3166_MOESM1_ESM.docx]
